# Mutational Landscape of *KIT* Proto-Oncogene Coding Sequence in 62 Canine Cutaneous and Subcutaneous Mast Cell Tumors

**DOI:** 10.3390/vetsci11120593

**Published:** 2024-11-25

**Authors:** Ludovica Montanucci, Elena Guidolin, Rosa Maria Lopparelli, Greta Mucignat, Marianna Pauletto, Mery Giantin, Mauro Dacasto

**Affiliations:** 1 Department of Neurology, McGovern Medical School, UTHealth—University of Texas Health Science Center at Houston, Houston, TX 77030, USA; ludovica.montanucci@uth.tmc.edu; 2 Department of Comparative Biomedicine and Food Science, University of Padua, Viale dell’Università 16, I-35020 Legnaro, Italy; elena.guidolin.000@gmail.com (E.G.); rosa.lopparelli@unipd.it (R.M.L.); greta.mucignat@phd.unipd.it (G.M.); marianna.pauletto@unipd.it (M.P.); mauro.dacasto@unipd.it (M.D.)

**Keywords:** canine mast cell tumors, *KIT* proto-oncogene, single nucleotide variants, internal tandem duplications

## Abstract

Canine mast cell tumors (MCTs) are common skin neoplasms with varying biological behaviors. The *KIT* proto-oncogene plays a key role in the development of these tumors, and internal tandem duplications on exon 11 are usually associated with more aggressive behavior, increased local recurrence, and decreased survival time. However, apart from exons 8–11 and 17, there is limited understanding of the overall *KIT* mutational landscape in canine MCTs. This work aims to analyze the entire *KIT* coding sequence (21 exons) in a cohort of 62 MCTs, which included 38 cutaneous and 24 subcutaneous tumors, and potentially identify new variants. In addition to confirming previously reported activating *KIT* mutations in exons 8, 9, and 11, we identified new variants in exons 2, 3, 5, 16, and the 3′ untranslated region (UTR). Notably, these last variants include an amino acid change (Asp/His) in exon 16. Additionally, we confirmed a differential prevalence of *KIT* variants in cutaneous and subcutaneous MCTs. These findings enhance our understanding of the *KIT* proto-oncogene coding sequence and provide valuable information for future confirmatory studies.

## Simple Summary

Canine mast cell tumors (MCTs) are the more prevalent form of skin tumor in dogs. Variants in a gene named *KIT*, coding for a transmembrane type III tyrosine kinase receptor, have been identified in these tumors and are likely to be disease-causing. These variants have been found to cause an excessive activation of the c-kit receptor, which, in turn, results in uncontrolled cell proliferation. Knowledge of the *KIT* variants is useful for understanding the molecular mechanism at the base of the disease, guiding targeted treatment, and for prognostic purposes. While many *KIT* variants have already been identified in exons 8–11 and 17, the full spectrum of canine MCT variants in both cutaneous and subcutaneous subtypes is not yet complete. Here, we collected 62 cutaneous and subcutaneous MCT samples from 56 dogs from 21 breeds, sequenced the full coding sequence of the *KIT* gene, and identified its variants. We confirmed previously known variants on exons 8–11 and identified new ones in subcutaneous MCT. Additionally, we confirmed the differential distribution of *KIT* variants between cutaneous and subcutaneous MCTs. Overall, we expanded the knowledge on the *KIT* mutational spectrum of these tumor types.

## 1. Introduction

The *KIT* proto-oncogene encodes a transmembrane type III tyrosine kinase receptor, which is involved in multiple signaling pathways that regulate cell proliferation, apoptosis, and differentiation [1]. This receptor is activated by the stem cell factor (SCF), which binds to the extracellular segment of the enzyme, comprising five immunoglobulin-like domains. Upon SCF binding, the enzyme undergoes dimerization and subsequent activation. The kinase activity is performed by the intracellular tyrosine–kinase domain (TKD), which is comprised of a phosphotransferase (PTD) and an ATP binding sub-domain [2].

Variants in the *KIT* gene have been documented in a variety of human and animal tumors. Gain-of-function mutations (i.e., mutations that cause the constitutive activation of c-kit tyrosine kinase) are considered a hallmark of human mastocytosis, and they have been proposed as a possible oncogenic mechanism for other types of human tumors, such as gastrointestinal stromal tumors, core-binding factor acute myeloid leukemias, melanomas, and seminomas (reviewed in [2]), as well as for canine and feline mast cell tumors (MCTs; reviewed in [3,4]). Activating mutations typically lead to constitutive c-kit phosphorylation and downstream activation independently from SCF binding [1].

In humans, the most common *KIT*-activating mutation found in more than 80% of adults with systemic mastocytosis is D816V in exon 17 of the TKD2 [5,6]. This variant has been proposed as a driver lesion for the disease, and it has been associated with worse prognosis and primary imatinib resistance [7,8]. Additionally, *KIT* mutations involving codon 560 in exon 11 and codons 815, 816, and 820 in exon 17 have been described in adult systemic mastocytosis, while E839K has been reported in pediatric mastocytosis [9].

In canine MCT, the most common cutaneous tumor in dogs, accounting for about 14% to 24% of all diagnosed canine skin tumors [10,11,12,13,14], mutations in the *KIT* gene occur, on average, in approximately 15% of canine cutaneous MCT (cMCT), with an increased incidence up to 35% in higher grade MCT [15]. Typically affecting older dogs, MCTs also occur in younger ones and particularly in breeds such as Boxers, Golden Retrievers, Pugs, Boston Terriers, and Pit Bull Terriers, which are more predisposed to this neoplasm. MCTs can manifest as solitary or multiple masses in cutaneous or subcutaneous tissues and show different biological behaviors, ranging from slow-growing, benign lesions to aggressive, ulcerated tumors that may metastasize [4,16].

The variants identified in canine MCT samples mostly involve *KIT* exon 11 [17,18,19,20], which codes for the intracellular juxtamembrane segment of the protein that, in the inactive state, maintains the two lobes of the kinase domain in a closed conformation [21], and exon 8, which, along with exon 9, codes for the extracellular immunoglobulin-like domain closest to the membrane [1]. A spectrum of changes, such as single nucleotide variants (SNVs), in-frame deletions, and internal tandem duplications (ITDs), have been identified in *KIT* exon 11. When variants in this exon were investigated through heterologous transfection, a ligand-independent activation of the enzyme, specifically a constitutive phosphorylation and an activation independent of SCF binding, was observed, indicating a gain-of-function effect of these variants as a possible disease mechanism [22]. In exon 8, ITDs of nucleotides 417–421 or the point mutation Q430R were observed in MCTs [21]. In exon 9, the first two reported missense mutations were S479I and N508I [18,21].

In canine MCT, the mutational profile of the *KIT* gene is associated with prognosis. Indeed, ITDs in exon 11 were associated with more aggressive tumor behavior, increased local recurrence, and decreased survival time [15,22,23,24] and have been proposed as prognostic indicators of MCT outcome [3]. Conversely, ITD in exon 8 was associated with longer survival and a milder course of the disease [25].

Interestingly, the mutational profile of the *KIT* gene has also been found to modulate drug responsiveness. In particular, tyrosine kinase inhibitors (TKIs) such as imatinib mesylate, which are the most used targeted therapies for human systemic mastocytosis, have proven to be ineffective when variant D816V was present [8]. In dogs, the two published registration trials for the TKIs toceranib phosphate and masitinib showed a higher objective response rate and a longer time to progression in patients with *KIT* gene mutations, respectively, although long-term outcomes were not reported [26,27,28]. Therefore, a comprehensive understanding of the *KIT* mutational profile is essential for informed therapeutic decision-making.

While there is abundant evidence of *KIT* mutations in exons 8, 9, and 11 being disease-causing, the full mutational landscape of the canine *KIT* gene in cutaneous and subcutaneous MCTs is only partially resolved; indeed, *KIT* whole exon sequence has been recently explored using an NGS approach (WES, whole exome sequencing) in only a small number of canine MCTs [29].

Thus, this study aims to expand knowledge on the canine exonic mutational landscape of *KIT* proto-oncogene by sequencing 62 cMCTs and subcutaneous MCTs (scMCTs) from a cohort of 56 canine individuals from 21 breeds. We aim to identify new potential variants in addition to the well-known ones located on exons 8–11 and 17 and, in silico, predict their potential pathogenicity. This work will help delineate the mutational landscape of the *KIT* gene, which is essential for understanding the molecular mechanism of the disease and guiding targeted treatments.

## 2. Materials and Methods

### 2.1. Sample Collection

For this retrospective molecular study, a total of 62 archived canine MCTs from 56 dogs, obtained either via fine needle aspiration (FNA) or biopsy, were analyzed. The cohort of samples comprises 38 cMCTs and 24 scMCTs. Overall, these samples were submitted to the Department of Comparative Biomedicine and Food Science at the University of Padua between 2012 and 2019 for the assessment of *KIT* mutational status. The signalment (breed, gender, age) of each patient was recorded and reported in Table S1. Kiupel histological grade [30] was available for 33 out of 38 cMCTs (Table S1).

Additionally, the two canine MCT cell lines most commonly used in in vitro studies, i.e., the C2 (from Centre de Recherche en Cancérologie, Marseille, France) and the NI-1 (a kind gift from the Medizinische Universität, Vienna, Austria) cell lines were considered. The two cell lines were grown as previously reported [31].

### 2.2. Total RNA Extraction

Total RNA extraction was performed using TRIzol™ Reagent (Thermo Fisher Scientific, Waltham, MA, USA) following the manufacturer’s instructions. The aqueous phase containing the RNA was then purified using the RNeasy^®^ Mini Kit (Qiagen, Venlo, The Netherlands). The quality of the extract was assessed using the NanoDrop^®^ ND-1000 Spectrophotometer (Thermo Scientific, Greenville, NC, USA). For downstream analyses, a minimum RNA concentration of 20 μg/mL was deemed adequate.

### 2.3. Reverse Transcription

Reverse transcription to synthesize complementary DNA (cDNA) was executed using the High-Capacity cDNA Reverse Transcription Kit (Applied Biosystems, Courtaboeuf, France), as reported by the manufacturer. One or 1.5 μg total RNA from FNA and biopsy samples, respectively, were loaded in each reaction (final reaction volume of 20 μL). The resulting cDNA was then stored at −20 °C until further analysis.

### 2.4. PCR Amplification

The complete coding sequence (cds) of the canine *KIT* gene was amplified by end-point PCR. Based on the Ensembl *KIT* transcript reference sequence ENSCAFT00030034940.1 (Genome assembly GCA 004886185.1, Ensembl release 113—October 2024), the coding sequence and flanking portions of the 5′ and 3′ untranslated regions (UTRs) were divided into four distinct amplicons. Each amplicon was generated using specific forward and reverse primers. Detailed information on the primers used is provided in Table S2. Amplicon #1 encompasses a segment of the 5′-UTR (−63 bp from ATG) and exons 1 through 5, while amplicon #2 covers exons 4 through 11. Amplicon #3 includes exons 9 through 17, and amplicon #4 extends from exons 16 to 21, including a portion of the 3′-UTR (201 bp from the stop codon). As the end of each amplicon overlaps with the beginning of the subsequent one, complete coverage and accurate sequencing of all exons are ensured.

PCR reactions were prepared in a total volume of 35 μL, consisting of 1.5 μL of diluted cDNA, 16.5 pmol of each primer, 0.7 U of Phire Hot Start II DNA Polymerase (Thermo Fisher Scientific, Waltham, MA, USA), 200 µM deoxynucleoside triphosphate mix (dNTPs), and 1X Phire Reaction Buffer (final concentrations). The thermal cycling conditions were as follows: (i) initial denaturation at 98.0 °C for 40 s; (ii) 42 cycles of denaturation at 98.0 °C for 6 s, annealing at 53–56 °C for 7 s, and extension at 72.0 °C for 15 s; (iii) final elongation at 72 °C for 1 min and 30 s. The annealing temperature was adjusted according to the melting temperature of the primers. Specifically, it was set at 53 °C for amplicon #1, 56 °C for amplicon #2, and 54 °C for both amplicons #3 and #4.

The amplified PCR products (at least two for each sample to address potential amplification errors) were pooled and visualized using gel electrophoresis on a 2% TBE agarose gel, followed by staining with EuroSafe Nucleic Acid Staining Solution (Euroclone, Milan, Italy). A 100-bp DNA ladder (Sharpmass 100-DNA ladder, EuroClone, Milan, Italy) served as the molecular weight marker. All amplified samples were subsequently stored at −20 °C for future analysis.

### 2.5. Amplicon Purification and Sequencing

The purification of pooled PCR amplicons (at least two PCR reactions per sample) was achieved through the Agencourt AMPure XP PCR Purification Kit (Beckman Coulter SRL, Milan, Italy), following the manufacturer’s instructions. After the purification, all *KIT* amplicons were quantified to ensure appropriate concentrations for sequencing. About 30–90 ng of DNA were provided to BMR Genomics (Padua, Italy) for the following Sanger sequencing.

### 2.6. Sequence Analysis

The chromatograms obtained from Sanger sequencing were analyzed using Chromas Lite software v.2.6.6 (Technelysium Pty Ltd., South Brisbane, QLD, Australia). Single nucleotide variants (SNVs) were identified manually according to the double peaks at the same position in the chromatograms. The forward and reverse sequences of each PCR amplicon were compared to the reference sequence using the multiple-sequence alignment tool MultAlin (http://multalin.toulouse.inra.fr/multalin/ accessed on 20 November 2024). Only variants confirmed by at least two independent sequencing reactions were considered.

### 2.7. Computational Prediction of Pathogenicity for SNVs and Computation of the Local Structural Environment

The potential impact of the identified SNPs was assessed using two different software tools: Ensembl Variant Effect Predictor (VEP) v.112.0 and Fido-SNP, a webserver recently developed by the University of Padua (https://snps.biofold.org/fido-snp/ accessed on 6 September 2024) [32].

For each missense variant, we retrieved the residues that were located in its local structural environment, defined as a sphere with a radius of 5 Å centered on the mutated residue. The structural environment was computed through a custom Python script developed in-house, which used the Bio.PDB module of BioPython.

### 2.8. Statistical Analysis

We applied Fisher’s exact test to statistically test differences between the MCT subtype (cMCT or scMCT) and cMCT grade (low or high) and *KIT* mutational status, considering each variant independently. The online tool Easy Fisher Exact Test Calculator (https://www.socscistatistics.com/tests/fisher/default2.aspx accessed on 3 September 2024) was used for this purpose, with the statistical significance set at *p* < 0.05. After the test, we applied a Bonferroni correction for multiple testing.

## 3. Results

### 3.1. Patient Demographics and Tumor Characteristics

This study included 62 canine MCTs from 56 dogs, comprising 38 cMCTs and 24 scMCTs. Among these, six dogs had multiple MCTs (four cMCTs and two scMCTs), while the remaining ones had a single tumor each. The average age of these dogs was 8.3 years, ranging from 2 to 15 years. Sixteen out of 56 dogs were males (including six castrated), and 39 were females (including twenty-five spayed); the gender of one dog was not provided. This study encompassed twenty-one different breeds: 12 Labrador Retrievers; 7 Boxers; 5 Setters; and 2 each of Beagle; French Bulldogs; Golden Retriever; Belgian Malinois; Maltese; and Pinscher. Additionally, there were 12 other breeds with only one dog each, i.e., Alaskan Malamute, American Staffordshire Terrier, Bleu de Gascogne, Bovaro del Bernese, Chihuahua, Dogo Argentino, Dogue de Bordeaux, Greyhound, Lhasa Apso, Schnauzer, Shar Pei, and Weimaraner. Finally, there were also nine mixed-breed dogs and one dog of unknown breed.

Histologic grading data were available for 33 cMCTs. According to the Kiupel grading system, 21 tumors were classified as low-grade and 12 as high-grade.

### 3.2. Diversity and Frequency of KIT Gene Mutations in Canine MCT

**Figure 1:**
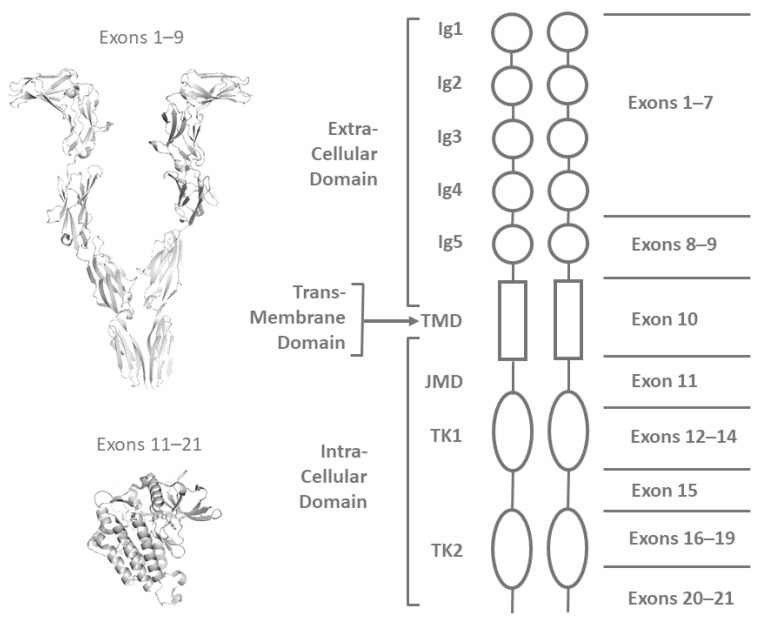
Graphical representation of the Type III tyrosine kinase receptor encoded by the *KIT* gene. Ig1–5 indicates the extracellular immunoglobulin-like domains; TMD indicates the transmembrane domain; JMD indicates the intracellular juxtamembrane domain; TK1 and TK2 indicate the tyrosine kinase domains. The PDB (Protein Data Bank) structures used for the visualizations are the structures of the human tyrosine kinase: 8DFP for the extracellular region (exons 1–9, top) and 7ZW8 for the intracellular region (exons 11–21, bottom).

Genetic alterations in the *KIT* coding sequence (see a schematic representation in Figure 1) were identified in all canine MCTs and were found to involve exons 2, 3, 5, 8, 9, 11, 12, 16, and the 3′ untranslated region (3′-UTR). Details of these mutations are summarized in Table 1. Both ITDs and SNVs were found.

#### 3.2.1. Exons 2 and 3

In exon 2, we identified one synonymous substitution, c.159C>T(p.Gly53=) in 16 out of 62 samples (25.8%). In exon 3, we identified three synonymous substitutions, namely, c.414C>T(p.Cys138=), c.507A>G(p.Lys169=) and c.621>A(p.Arg207=), in 6, 25, and 2 samples out of 62, respectively. All the variants identified in exons 2 and 3 are known germline variants cataloged in dbSNP and are identified with a rs-ID.

#### 3.2.2. Exons 4 and 5

At the boundary of exons 4 and 5 of the *KIT* gene, specifically in the NAGNAG splice acceptor site (i.e., CAGCAG), an in-frame deletion of the triplet CAG was observed in all 62 samples. This deletion (c.763_765del(p.Gln255del)) leads to the removal of a glutamine residue at position 255 along the protein sequence. Localized on a splicing site, it might give origin to two alternative transcripts. Interestingly, this glutamine residue is conserved in most mammal species whose *KIT* sequence has been curated in Uniprot (human, marmoset, mouse, dog, cat, cattle, pig, and goat.

#### 3.2.3. Exons 8 and 9

In exon 8, the well-known 12 bp-long internal tandem duplication (c.1251_1262dup) was identified in six samples (9.7%). This duplication causes the insertion of a glutamine residue followed by the duplication of the three residues Ile418, Leu419, and Thr420.

In exon 9, two non-synonymous SNVs, c.1436G>T(p.Ser479Ile) and c.1523A>T(p.Asn508Ile), were observed in 2 (3.2%) and 1 (1.6%) samples, respectively.

#### 3.2.4. Exons 11 and 12

In exon 11, we identified seven distinct in-frame ITDs, occurring in seven samples (11.3%), all partially overlapping and all located toward the 3′ end of the exon 11 between residues 570 and 590 of the c-kit protein. Of these, variant c.1737_1738insGAATGGG;1706_1737dup is a complex variant (potentially in a retrocopy) in which insertion of seven base pairs (GAATGGG) is immediately followed by a 32 bp-long duplication of the cDNA region from 1706 to 1737. This results in the insertion of three residues (glutamate, tryptophan, and aspartic acid) between the two copies of the duplication. Moreover, we identified a further ITD, c.1729_1791dup, spanning the 3′ end of exon 11 and the beginning 5′ end of exon 12, which affects amino acids from 577 to 597.

**Figure 2:**
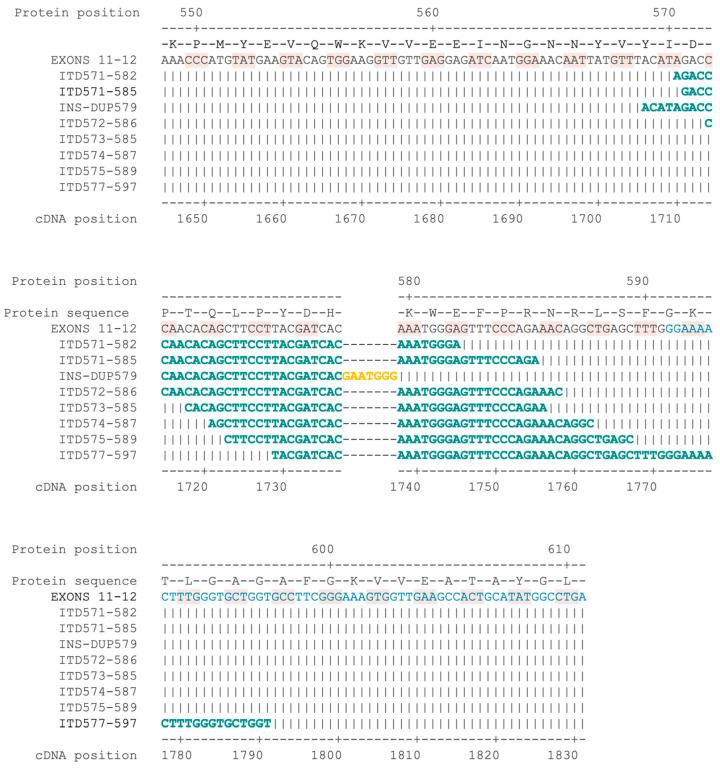
cDNA sequence of exons 11 and 12 and its protein translation. The black cDNA sequence indicates exon 11, while the blue cDNA sequence indicates exon 12. The individual codons are highlighted with alternating clear and pink shadows. The ITDs and the complex insertion–duplication are represented in each subsequent line. For each variant, the sequence of only the duplicated region is reported (bold, green). The sequence highlighted in yellow corresponds to the 7 bp insertion of INS-DUP579.

An image showing the alignment of the different insertions identified in the present cohort of MCTs is reported in Figure 2. All the ITDs identified in exons 11 and 12 in this study had been previously reported, except for the complex variant c.1737_1738insGAATGGG;1706_1737dup.

In exon 11, we also identified two SNVs: a non-synonymous SNV, c.1673T>G(p.Val558Gly), in one sample (1.6%) and a synonymous SNV c.1731C>T(p.Tyr577=) in 17 samples (27.4%).

#### 3.2.5. Exon 16

In exon 16, two SNVs were identified: a non-synonymous SNV, c.2299G>C(p.Asp767His), and a synonymous SNV, c.2355G>A(p.Lys785=). The first one was observed in two samples (3.2%), while the second one was observed in one sample (1.6%). Variant c.2299G>C(p.Asp767His) is reported here for the first time as it has not been previously documented in the literature.

#### 3.2.6. 3′-UTR Region

Two SNVs, c.2965G>T and c.3036G>A, were identified in the 3′-UTR, which is 198 bp long. Both variants are located in the first half of the 3′-UTR, with variant c.2965G>T located only 24 bases from the end of exon 21. Both SNVs are known variants cataloged in dbSNP and endowed by a SNP identifier (rs-ID).

### 3.3. Computational Prediction of Pathogenicity for the Identified SNVs

We assessed the potential functional impact of the identified SNVs using the SIFT method provided in the VEP package and the Fido-SNP method, which is a binary classifier specifically optimized for SNV dog variants. While SIFT-VEP only scores non-synonymous variants, Fido-SNP provides pathogenicity prediction for both synonymous and non-synonymous variants. The pathogenicity predictions from these two tools are provided in Table 1. Out of the 12 SNVs identified in our samples, all four non-synonymous substitutions were predicted to be pathogenic by both methods. Out of the six synonymous substitutions, two were predicted to be pathogenic, and the remaining four were predicted to be benign by Fido-SNP. Of the two SNVs present in the 3′-UTR, c.2965G>T is predicted to be benign, while c.3036G>A is predicted to be pathogenic.

Out of the four non-synonymous variants, two are located in exon 9 and the structural region of the fifth extracellular immunoglobulin-like C2 type 5 domain. Variant p.(Val558Gly) is located in exon 11 and the intracellular juxtamembrane domain, while p.(Asp767His) is located in exon 16 and the protein kinase domain. None of these residues is involved in any binding domain.

### 3.4. Computation of the Local Structural Environment

For each missense variant, we analyzed the structural environment of its wild-type residue. Using the PDB structure 7ZW8 of the human tyrosine kinase, we found that residue Val558 (of variant Val558Gly) is located at less than 5 Å from a Tyrosine residue (Tyr570 in human and Tyr569 in canine *KIT*), which serves as major phosphorylation site for the enzyme activation. For the other three missense variants, their structural local environment does not contain residues with known critical functions.

### 3.5. Full KIT Sequencing in C2 and NI-1 Canine MCT Cell Lines

We applied the same experimental approach (*KIT* full-sequencing from cDNA) to the C2 and the NI-1 cell lines to complete and confirm, respectively, the mutational profiling of *KIT* cds. While for the NI-1 cells, the full sequence of all exonic regions was available [33], for the C2 cells, only exons 9–12 had been sequenced [34]. The variants identified in these two cell lines are shown in Table 2.

In the C2 cell line, we confirmed a 48 bp-long insertion, causing a complex indel at the protein level [34]; we also identified additional SNVs located on exons never screened before (i.e., c.159C>T, c.414C>T, c.507A>G, and c.3036G>A in exons 2, 3, and the 3′-UTR, respectively).

In the NI-1 cell line, we confirmed the ITD417–421 in exon 8, as well as the synonymous SNVs in exon 2 (c.107C>T), exon 3 (c.414C>T, and c.507A>G), and exon 7 (c.1187A>G) [33].

Both cell lines exhibit c.414C>T and c.507A>G synonymous SNVs and the c.763_765del CAG deletion at the 5′-end of exon 5, which was never reported in previous publications.

Finally, these cell lines harbor variants that were not present in the 62 samples of this study, specifically the two SNVs c.107C>T and c.1187A>G, and the 48 bp-long insertion in exon 11.

### 3.6. KIT Mutation Frequency in cMCTs and scMCTs

As a whole, 18 MCTs out of 62 showed *KIT*-activating mutations (indels or non-synonymous deleterious SNVs) in the canonical exons 8, 9, and 11, but no MCT had more than one *KIT*-activating mutation in these exons. Conversely, the majority of cases showed more than one variant (mostly synonymous), considering the entire cds.

The genotyping results in cMCT and scMCT samples, separately, are summarized in Table 3.

In scMCTs, no ITD was found in exons 11–12, while eight different ITDs were identified in cMCTs. Conversely, a higher prevalence of ITD417–421 was observed in scMCTs (16.7%) when compared to cMCTs (5.3%).

Synonymous variants in exons 2, 3, and 11, as well as the ones in 3′-UTR, were present in both cMCT and scMCT samples, while all the SNVs of exon 9 were exclusively found in scMCTs (*n* = 3). The deleterious missense SNV of exon 11 was observed in one cMCT case only. Overall, the non-synonymous SNVs identified in exons 9, 11, and 16, which were predicted to be deleterious, were more frequent in scMCTs (*n* = 5) than cMCTs (*n* = 1). Moreover, the deletion of the glutamine at position 255 of the protein sequence was found in all cMCT and scMCT samples.

We investigated for each variant, separately, the different prevalence between cMCTs and scMCTs with Fisher’s Exact test, but we found no statistical significance after Bonferroni correction.

### 3.7. KIT Mutation Frequency and cMCT Histological Grade

Out of the 38 cMCTs analyzed in this study, 33 were graded using the Kiupel histological grading system [30], while for the remaining five samples, the grade was not provided. We distinguished 21 low-grade and 12 high-grade MCTs. A summary of the mutations found in cMCT samples according to the grading is provided in Table 4.

In the cohort considered in the present study, ITD417–421 was recorded in low-grade cMCTs only; conversely, indels of exons 11–12 appeared more frequent in high-grade cMCTs (41.7%) compared to low-grade ones (14.3%). A similar distribution (a higher prevalence in high-grade cMCTs) was also observed for the pathogenic synonymous SNVs c.159C>T and c.3036G>A. As to benign synonymous SNVs and the deletion of exon 5, they were commonly detected in both low-grade and high-grade cMCT samples.

We investigated these differences, along with differences for each variant, separately, with Fisher’s Exact test, but no statistically significant differences were found after Bonferroni correction, likely due to the small number of samples.

## 4. Discussion

In this work, we expanded the knowledge about the mutational landscape of the *KIT* gene by sequencing its entire coding sequence in 62 MCTs from 56 dogs. While most studies have focused solely on variants in exons 8, 9, and 11, recent NGS sequencing of the entire gene has been conducted on 48 MCTs [29]. However, the MCT cohort of the NGS study included only a small number (*n* = 7) of subcutaneous samples [29]; therefore, there is still no sufficient data to draw conclusive generalizations about *KIT* mutational status in scMCT. To this purpose, in this work, we present Sanger sequencing data for all 21 exons of the *KIT* gene from 62 MCT samples, consisting of a balanced representation of cutaneous (*n* = 38) and subcutaneous (*n* = 24) cases. In particular, while Chen and collaborators [35] previously processed more than 200 scMCT samples, restricting the analysis to exons 8–11, our study provides the full coding sequence of *KIT* for the largest number of scMCTs to date. Overall, this work confirms that most variants associated with the disease are located in exons 8, 9, and 11. Nevertheless, we also identified variants in other exons (i.e., 2, 3, 5, 12, 16, and 3′-UTR) and evaluated their potential impact on c-kit receptor function through a bioinformatic analysis and a computational prediction.

The ITDs in exon 11 are known to be associated with the constitutive phosphorylation of c-kit and are among the most studied alterations in the *KIT* coding sequence. They are also the most prevalent, occurring in approximately 20% of canine MCTs [15]. In this study, we identified ITDs in exon 11 in 12.9% of cases (8 out of 62) when considering the entire cohort and 21% (8 out of 38) when considering only cMCTs. The prevalence in the cutaneous subtype observed in this study was comparable to that reported in previous studies [11]. More specifically, we confirmed the high variability of ITDs by identifying eight different ITDs (with one ITD extending into exon 12), all involving amino acid residues 571–590. Notably, residues 567 and 569, which are both tyrosines and correspond to tyrosines 568 and 570 in the human protein, serve as principal autophosphorylation sites that trigger the dimerization and subsequent activation of the receptor [36]. All the ITDs reported here have been previously described [18,21], except for a novel genetic alteration, a 7-bp insertion followed by a duplication, which leads to the insertion of three amino acids—glutamine, tryptophan, and aspartic acid—before the duplication. These complex variants underscore the high variability of this exon in MCTs.

ITDs in exon 11 were found to be more abundant in cMCTs, occurring in about 20% of cases [25], compared to scMCTs, where they are rarely observed [37]. A recent retrospective study of 216 scMCTs detected ITDs in exon 11 in 12 out of 216 scMCT cases (5.6%) [35]. In the present study, we confirm the prevalence of ITDs in exon 11 in cMCTs, even if with no statistical significance. This finding aligns with the concept that scMCTs are generally considered less aggressive than their cutaneous counterparts [38]. Among cMCTs, we observed a higher prevalence of these gain-of-function variants in high-grade compared to low-grade cMCTs (41.7% vs. 14.3%). Despite the lack of statistical significance due to the limited number of cases, this result is consistent with the current literature. Several studies have similarly reported increased frequencies of ITDs and deletions of exon 11 in higher histologic grade MCTs [15,39,40,41]. Indeed, MCTs with exon 11 indels have been significantly associated with increased cellular proliferation markers [41,42], higher recurrence and metastasis rates [39], and reduced overall survival [23,42].

In addition to ITDs, a known SNV in exon 11 was also identified in our cohort, specifically in one cutaneous case only. This missense variant, Val558Ile, has been previously reported [21] and is predicted to be pathogenic by Fido-SNP. As noted by Letard and collaborators [21], Val558 is located less than 5 Å from one of the two tyrosine residues in the juxtamembrane domain that serve as phosphorylation sites, regulating the stability of the inactive conformation of c-kit and its activation. Therefore, variants at Val558 may disrupt this regulatory function.

In exon 8, we confirm a recurrent 12 bp-long ITD (ITD417–421), which has frequently been found in canine MCTs [15,18,21,25,43,44]. Unlike exon 11 ITDs, the insertion in exon 8 was most commonly found in MCTs that caused only local disease and has not been associated with poor prognosis [25]. Likewise, in the present study, this insertion has been detected only in cMCT samples classified as Kiupel low-grade. Regarding scMCTs, the prevalence of ITD417–421 is higher in subcutaneous samples compared to cutaneous ones (16.7% and 5.3%, respectively), although the low number of samples prevents statistical significance. ITD417–421 was the only variant identified in scMCT by Vozdova and collaborators [29] and one of the few (*n* = 3; one ITD in exon 8 and two deleterious missense mutations in exon 9) detected by Marconato and colleagues [45]. A recent paper by Chen and colleagues reported the well-known ITD variant of exon 8 in 10.6% of scMCT cases (23 out of 216), with a higher prevalence in Labrador Retrievers [35].

In exon 9, which encodes part of the receptor’s ligand binding domain, we identified two missense SNVs, c.1436G>T(p.Ser479Ile) and c.1523A>T(p.Asn508Ile), both first reported in cMCT by Letard and collaborators [21]. Notably, in the present study, these variants were observed only in scMCTs (*n* = three cases). This finding is significant for our understanding of scMCT, as missense SNVs in this MCT subtype have been previously described only by the same authors [45]. Therefore, a deeper understanding of the role these SNVs play in canine scMCT might be valuable, particularly in investigating whether an association exists with the scMCT cases that exhibit more aggressive and unpredictable behavior. Indeed, as hypothesized in previous studies, variants in the fifth immunoglobulin-like domain might enhance the binding affinity to and within this domain, leading to the activation of the intracellular domain [21,46].

Among the exons previously investigated in canine mastocytoma, exon 17 was also considered [21]. In humans, this exon represents a hotspot for activating mutations, especially the well-known D816V substitution [47], which is considered the hallmark of human adult mastocytosis [48]. In the present study, no mutations were observed in exon 17. This absence may suggest that this coding region does not harbor mutations linked to canine MCT pathogenesis and development, or it might simply reflect differences in the considered canine population.

Following the Oncology–Pathology Working Group, a joint initiative of the Veterinary Cancer Society and the American College of Veterinary Pathologists, that recently promoted additional investigations on mutations located in various exons, further than in exons 8 and 11 [15], this study focused on the entire *KIT* coding sequence. The results obtained for exons 1–7, 13–16, 18–21, and partial 3′-UTR are discussed below.

All variants identified in exons 2 and 3, as well as in the 3′-UTR of our samples, are cataloged in dbSNP, suggesting that they are more likely to be germline polymorphisms rather than variants associated with MCT. While two of these variants are predicted to be pathogenic by Fido-SNP, this prediction contrasts with their presence in dbSNP, which typically indicates benign polymorphisms. Therefore, none of these SNVs are strong candidates for being disease-causing. Notably, they were similarly distributed across both cMCTs and scMCTs, as well as among low-grade and high-grade cMCTs. To support this hypothesis, future comparative *KIT* sequencing of both tumor core and surgical margins will be required.

In exon 5, we identified an in-frame deletion (c.763.765del) of one codon (CAG) in all 62 samples, that is, in 100% of both cMCTs and scMCTs of our study. This variant had been previously reported in canine MCTs with a prevalence of 44.7% [18]. However, it was not observed in the 48 MCTs analyzed in the NGS study [29]. This deletion, which causes the loss of a glutamine residue at position 255, has also been identified in canine healthy tissues (cerebellum [34] and renal cortex [49]). Deeper analyses of this variant showed that c.763.765del was located at the boundary of exons 4 and 5 of the *KIT* gene, specifically in the NAGNAG splice acceptor site; thus, it might be responsible for the origin of two alternative transcripts, as already reported in the Chinese raccoon dog (*Nyctereutes procyonoides procyonoides*) [50]. To support this hypothesis, we aligned the raw RNA-seq reads we obtained from previous investigations on the C2 cell line (four biological replicates, unpublished data) to the reference cDNA sequence ENSCAFT00030034940.1. This approach allowed us to estimate the gene expression of the allele harboring CAG deletion, which in the cell line was ~30–32%, and confirm the presence of the two alternative transcripts.

Structurally, this deletion is located at the end of an eight-residue unstructured loop that connects two beta-sheets in the extracellular domain. This loop is not a known functionally significant region of the protein. Among the available mammalian c-kit protein sequences curated in Uniprot (human, marmoset, mouse, dog, cat, cattle, pig, and goat), this residue was conserved in all species except for the cat, where the same deletion was present, and the mouse, which showed a proline insertion near the deleted glutamine. Thus, in cats and mice, the loop length connecting the two beta-sheets is seven and nine residues, respectively, suggesting that a one-residue difference in loop length does not compromise the protein function. Furthermore, the presence of this deletion in the reference genome of a related species (cat) supports its likely non-disruptive nature, suggesting that it may be a common variant in the dog genome. Nevertheless, although Glu255del is likely benign, it may still affect susceptibility to MCT; further data is necessary to assess this possibility and determine whether this variant is differently distributed among dog breeds.

In exon 16, we identified two SNVs, of which the non-synonymous one (Asp767His) is reported here for the first time. It is worth mentioning that it was observed in only two scMCT cases. This variant is located in the intracellular kinase domain TK2 and is predicted to be pathogenic by Fido-SNP. When we investigated its local structural environment, we found that this variant did not interact with any known functional sites. Nevertheless, its functional impact should be further investigated in vitro through heterologous expression. The deepening of its function might expand the knowledge on the molecular basis and the clinical behavior of canine scMCT.

In addition to the aforementioned variants, we identified synonymous benign SNVs in exons 11 and 16, specifically c.1731C>T and c.2355G>A, in 27.4% and 1.6% of MCT cases, respectively. These polymorphisms were already known [21].

Finally, we analyzed the two canine MCT cell lines, NI-1 and C2, which are commonly used in canine mastocytoma research. For the NI-1 cells, we confirmed all previously identified exonic mutations, including the missense mutations at nucleotides 107 and 1187 of the cDNA and a 12-bp duplication at nucleotide 1263, as reported by the authors who established and characterized the cell line [33]. Additionally, we observed the deletion of the triplet CAG at codon 255, a splice variant not previously reported. For the C2 cell line, for which only data about *KIT* exons 9–12 were available up to date, we confirmed the 48 bp-long insertion in exon 11 [34] while also providing a more comprehensive molecular characterization by identifying four synonymous SNVs on exons 2–3 and 3′-UTR (i.e., c.159C>T, c.414C>T, c.507A>G and c.3036G>A) and the deletion c.763_765del in exon 5. We did not observe the 12 bp-long deletion described by London and colleagues in exon 9 [34], probably due to the dissimilarity between the two reference sequences used for the alignment (ENSCAFT00030034940 and GenBank no. AF099030).

A limitation of this work is that variants located in non-coding regions could not be detected due to the specific experimental design of this study. Indeed, by sequencing amplicons generated by end-point PCR amplification of *KIT* cDNA, intronic regions were not sequenced despite their potential functional relevance. To fully capture the *KIT* mutational landscape in canine MCT, it would be valuable in future studies to include both intronic and splicing variants. Additionally, evaluating the expression levels of every single genetic mutation, also in association with the histological grade of the tumor, could provide insights into their significance for disease progression.

Additionally, the 56 MCT-bearing dogs (16 males and 39 females) selected for this study were sourced from the tissue archive of a diagnostic laboratory specialized in the *KIT* mutational analysis (exons 8, 9, and 11) in canine MCT for veterinary practitioners. The selection was primarily based on the availability of a sufficient total RNA to cover the entire experimental analysis, which may account for the unbalanced gender distribution. Notably, there is no conclusive evidence supporting sex-based predisposition for MCT development to date [10,11,51,52].

As a whole, the association analysis between the *KIT* mutation spectrum and cMCT severity (as measured by the Kiupel grading system) did not yield significant findings despite a reasonable sample size. This outcome likely reflects the high diversity of mutation types observed within the *KIT* gene, resulting in limited statistical power. These findings suggest that multiple, varied pathways may lead to MCT development, underscoring the need for large-scale meta-analyses to determine which specific mutations are causative in this form of cancer.

## 5. Conclusions

The present study expands the knowledge on the mutational landscape of the c-kit canine protein, particularly for scMCTs, through the sequencing of 38 cMCTs and 24 scMCTs. We confirm that ITDs in exon 11 are the most prevalent variants, and we found that they are more abundant in cMCTs compared to scMCTs, and, among cMCTs, they have a higher prevalence in Kiupel high-grade compared to low-grade cMCTs. Conversely, ITDs in exon 8 are more prevalent in scMCTs than in cMCTs. Non-synonymous variants in exon 9 were only found in scMCTs. Finally, a novel SNV in exon 16, Asp767His, located in the intracellular kinase domain TK2, was identified in two scMCTs. This variant is in silico predicted to be pathogenic using the Fido-SNP computational tool.

In summary, this work delineates two different mutational profiles for cMCTs and scMCTs, with cMCTs characterized by variants (ITDs and SNVs in exon 11) associated with the severity of the disease and scMCTs characterized by less impacting ones (ITDs in exon 8). Nevertheless, scMCTs’ mutational profiles appeared to be also characterized by a higher prevalence of deleterious SNVs in exons 9 and 16, whose role in the biology of the tumor should be deepened to potentially clarify its debated clinical behavior.

Overall, these results provide new insights into the *KIT* proto-oncogene coding sequence and the occurrence of mutations in canine cMCTs and scMCTs.

## Figures and Tables

**Table 1:** List of *KIT* variants identified in 62 canine MCT samples.

Exon	cDNA Variant	Protein Variant	rs-ID	ITD Name	Samples (*n*, %)	VEP-SIFT	FidoSNP
2	c.159C>T	p.(Gly53=)	rs851679960	na	16/62 (25.8%)	na	Pathogenic
3	c.414C>T	p.(Cys138=)	rs850580536	na	6/62 (9.7%)	na	Benign
3	c.507A>G	p.(Lys169=)	rs852718113	na	25/62 (40.3%)	na	Benign
3	c.621G>A	p.(Arg207=)	rs852786648	na	2/62 (3.2%)	na	Pathogenic
5	c.763_765del	p.(Gln255del)	na	na	62/62 (100.0%)	na	na
8	c.1251_1262dup	p.(Thr420_His421insGln; Ile418_Thr420dup)	na	ITD417–421	6/62 (9.7%)	na	na
9	c.1436G>T	**p.(Ser479Ile)**	na	na	2/62 (3.2%)	Deleterious	Pathogenic
9	c.1523A>T	**p.(Asn508Ile)**	na	na	1/62 (1.6%)	Deleterious	Pathogenic
11	c.1673T>G	**p.(Val558Gly)**	na	na	1/62 (1.6%)	Deleterious	Pathogenic
11	c.1731C>T	p.(Tyr577=)	rs853024368	na	17/62 (27.4%)	na	Benign
11	c.1710_1745dup	p.(Asp571_Glu582dup)	na	ITD571–582	1/62 (1.6%)	na	na
11	c.1711_1755dup	p.(Asp571_Arg585dup)	na	ITD571–585	1/62 (1.6%)	na	na
11	c.1737_1738insGAATGGG;1706_1737dup	p.(His579_Lys580insGluTrpAsp; Ile570_579dup)	na	na	1/62 (1.6%)	na	na
11	c.1714_1758dup	p.(Pro572_Asn586dup)	na	ITD572–586	1/62 (1.6%)	na	na
11	c.1718_1756dup	p.(Thr573_Arg585dup)	na	ITD573–585	1/62 (1.6%)	na	na
11	c.1721_1762dup	p.(Gln574_Arg587dup)	na	ITD574–587	1/62 (1.6%)	na	na
11	c.1723_1767dup	p.(Leu575_Ser589dup)	na	ITD575–589	1/62 (1.6%)	na	na
11–12	c.1729_1791dup	p.(Tyr577-Gly597dup)	na	ITD577–597	1/62 (1.6%)	na	na
16	c.2299G>C	**p.(Asp767His)**	na	na	2/62 (3.2%)	Deleterious	Pathogenic
16	c.2355G>A	p.(Lys785=)	na	na	1/62 (1.6%)	na	Benign
3′-UTR	c.2965G>T	--	rs8793422	na	17/62 (27.4%)	na	Benign
3′-UTR	c.3036G>A	--	rs8793421	na	16/62 (25.8%)	na	Pathogenic

All the positions are annotated on the ENSCAFT00030034940.1 transcript (Genome assembly GCA 004886185.1, Ensembl release 113—October 2024). In the Protein Variant column, missense variants are highlighted in bold. When the SNV is cataloged in the dbSNP database, its rs-ID (Reference SNP ID) is reported. The duplicated cDNA sequences are as follows: c.1251_1262dup: AATCCTGACTCA; c.1710_1745dup: AGACCCAACACAGCTTCCTTACGATCACAAATGGGA; c.1711_1755dup: GACCCAACACAGCTTCCTTACGATCACAAATGGGAGTTTCCCAGA; c.1706_1737dup: ACATAGACCCAACACAGCTTCCTTACGATCAC; c.1714_1758dup: CCAACACAGCTTCCTTACGATCACAAATGGGAGTTTCCCAGAAAC; c.1718_1756dup: CACAGCTTCCTTACGATCACAAATGGGAGTTTCCCAGAA; c.1723_1767dup: CTTCCTTACGATCACAAATGGGAGTTTCCCAGAAACAGGCTGAGC; c.1721_1762dup: AGCTTCCTTACGATCACAAATGGGAGTTTCCCAGAAACAGGC; c.1729_1791dup: TACGATCACAAATGGGAGTTTCCCAGAAACAGGCTGAGCTTTGGGAAAACTTTGGGTGCTGGT.

**Table 2:** *KIT* variants identified in C2 and NI-1 canine cell lines.

C2 Cell Line			NI-1 Cell Line		
Exon	cDNA Variant	Protein Variant	Exon	cDNA Variant	Protein Variant
**2**	**c.159C>T**	**p.(Gly53=)**	2	c.107C>T	p.(Pro36Leu)
**3**	**c.414C>T**	**p.(Cys138=)**	3	c.414C>T	p.(Cys138=)
**3**	**c.507A>G**	**p.(Lys169=)**	3	c.507A>G	p.(Lys169=)
**5**	**c.763_765del**	**p.(Gln255del)**	**5**	**c.763_765del**	**p.(Gln255del)**
11	c.1756_1757ins **	p.Glu586delins ***	7	**c.1187A>G**	p.(Gln396Arg)
**3′-UTR**	**c.3036G>A**	--	8	c.1251_1262dup	p.(Thr420_His421insGln; Ile418_Thr420dup)

The variants highlighted in bold have been reported for the first time in the two cell lines. ** The insertion in exon 11 is c.1756_1757insCATACCCAACACAGCTTCCTTACGATCACAAATGGGAGTTTCCCAGA. *** Its predicted effect on the protein sequence is p.Glu586delinsAlaTyrProThrGlnLeuProTyrAspHisLysTrpGluPheProArgLys.

**Table 3:** *KIT* variant frequencies in 38 cMCTs and 24 scMCTs.

	Exon	Variant	cMCT (*n*, %)	scMCT (*n*, %)
ITD exon 8	8	ITD417–420	2/38 (5.3%)	4/24 (16.7%)
Indels exons 11–12	11–12	indels	8/38 (21.1%)	--
Non-synonymous SNV—deleterious	9	c.1436G>T(p.Ser479Ile)	--	2/24 (8.3%)
	9	c.1523A>T(p.Ans508Ile)	--	1/24 (4.2%)
	11	c.1673T>G(p.Val558Gly)	1/38 (2.6%)	--
	16	c.2299G>C(p.Asp767His)	--	2/24 (8.3%)
Synonymous SNV—pathogenic	2	c.159C>T(p.Gly53=)	7/38 (18.4%)	9/24 (37.5%)
	3	c.621G>A(p.Arg207=)	--	2/24 (8.3%)
	3′-UTR	c.3036G>A	7/38 (18.4%)	9/24 (37.5%)
Synonymous SNV—benign	3	c.414C>T(p.Cys138=)	3/38 (7.9%)	3/24 (12.5%)
	3	c.507A>G(p.Lys169=)	15/38 (39.5%)	10/24 (41.7%)
	11	c.1731C>T(p.Tyr577=)	11/38 (28.9%)	6/24 (25.0%)
	16	c.2355G>A(p.Lys785=)	--	1/24 (4.2%)
	3′-UTR	c.2965G>T	13/38 (34.2%)	4/24 (16.7%)
Deletion exon 5	5	c.763_765del(p.Gln255del)	38/38 (100.0%)	24/24 (100.0%)

The number and the percentage of samples harboring the variant are reported for each MCT subtype.

**Table 4:** *KIT* variant frequency and Kiupel histological grade.

	Exon	Variant	Mutated cMCT (*n*)	Mutated cMCT Not Graded (*n*)	Low-Grade (*n*, %)	High-Grade (*n*, %)
ITD exon 8	8	ITD417–421	2/38	--	2/21 (9.5%)	--
Indels exons 11–12	11–12	indels	8/38	--	3/21 (14.3%)	5/12 (41.7%)
Non-synonymous SNV—deleterious	11	c.1673T>G(p.Val558Gly)	1/38	1/1	--	--
Synonymous SNV—pathogenic	2	c.159C>T(p.Gly53=)	7/38	--	3/21 (14.3%)	4/12 (33.3%)
	3′-UTR	c.3036G>A	7/38	--	4/21 (19.0%)	3/12 (25.0%)
Synonymous SNV—benign	3	c.414C>T(p.Cys138=)	3/38	--	2/21 (9.5%)	1/12 (8.3%)
	3	c.507A>G(p.Lys169=)	15/38	5/15	5/21 (23.8%)	5/12 (41.7%)
	11	c.1731C>T(p.Tyr577=)	11/38	2/11	7/21 (33.3%)	2/12 (16.7%)
	3′-UTR	c.2965G>T	13/38	1/13	5/21 (23.8%)	7/12 (58.3%)
Deletion exon 5	5	c.763_765del(p.Gln255del)	38/38	--	21/21 (100.0%)	12/12 (100.0%)

The number and the percentage of low-grade and high-grade cMCT samples harboring each variant are reported.

## Data Availability

The original contributions presented in this study are included in the article/Supplementary Materials; further inquiries can be directed to the corresponding author.

## Supplementary Materials

The following supporting information can be downloaded at https://www.mdpi.com/article/10.3390/vetsci11120593/s1, Table S1: List of 62 canine MCT samples from 56 dogs; Table S2: Oligonucleotide primers used for canine *KIT* full sequencing.
