# Supplementary material for: Mutational Landscape of KIT Proto-Oncogene Coding Sequence in 62 Canine Cutaneous and Subcutaneous Mast Cell Tumors
Source: Vet Sci. 2024 Nov 25;11(12):593. doi: 10.3390/vetsci11120593 (PMC11680137; doi:10.3390/vetsci11120593)
Supplement: Supplementary file 1 [file vetsci-11-00593-s001.zip › vetsci-3274404-supplementary.pdf]

veterinary sciences

Table S1. List of 62 canine MCT samples from 56 dogs.

Sample	Breed	Gender	Age (yy)	MCT c/sc	Kiupel histological grade
1	Malinois	MC	3	sc	--
2	Greyhound	MC	5	sc	--
3	Boxer	F	6	c	low
4	Mixed breed	M	10	sc	--
5	Labrador retriever	F	14	sc	--
6	Labrador retriever	FS	8	c	high
7	Boxer	FS	10	sc	--
8	Dogo argentino	FS	9	c	NP
9A-9B	Boxer	FS	10	sc	--
10A-10B	English Setter	F	7	c	low
11	English Setter	M	7	c	low
12	Maltese	MC	10	sc	--
13	Pinscher	F	11	c	NP
14	Boxer	FS	10	c	low
15A-15B	Labrador retriever	FS	5	c	high
16	Setter	FS	9	c	NP
17	Lhasa Apso	MC	11	sc	--
18	Labrador retriever	FS	8	c	low
19	Mixed breed	FS	8	sc	--
20	Golden retriever	FS	8	sc	--
21	Labrador retriever	F	10	c	high
22	Setter gordon	M	9	sc	--
23	Labrador retriever	FS	7	c	high
24	Mixed breed	FS	7	sc	--
25	Labrador retriever	FS	11	c	low
26	Labrador retriever	M	6	c	low
27	Malinois	FS	7	sc	--
28	French Bulldog	MC	7	c	high
29	Labrador retriever	F	9	sc	--
30	Mixed breed	FS	2	c	high
31	Labrador retriever	M	8	c	low
32	Boxer	F	4	c	NP
33	Beagle	M	12	c	low
34	Weimaraner	F	7	c	low
35	Boxer	M	11	c	low
36	Bovaro del Bernese	FS	6	c	high

37	American Staffordshire terrier	FS	5	c	low
38	Mixed breed	MC	11	sc	--
39	Labrador retriever	FS	11	sc	--
40	Golden retriever	F	13	sc	--
41	Pinscher	F	9	c	high
42	Mixed breed	NP	6	c	high
43	Shar pei	F	10	c	low
44	Maltese	FS	15	c	low
45A-45B	Schnauzer	FS	8	c	low
46	Alaskan malamute	F	4	c	low
47A-47B	French Bulldog	M	8	c	low
48	Beagle	M	3	sc	--
49	Mixed breed	FS	11	c	high
50	Mixed breed	FS	8	sc	--
51	Chihuahua	FS	8	c	NP
52	Labrador retriever	F	8	sc	--
53	Bleu de Guascoine	FS	11	c	low
54	Boxer	F	13	c	high
55A-55B	Dogue de bordeaux	FS	7	sc	--
56	English Setter	M	6	sc	--

M: male; F: female; MC: male castrated; FS: female spayed; c: cutaneous; sc: subcutaneous; NP: not provided

Table S2. Oligonucleotide primers used for canine *KIT* full sequencing.

Amplicon	Primer Sequence (5′-3′)	Amplified region	Amplicon length (bp)
#1	F: TCCGTGCACTTGGGC<br>R: TTCCAAGGTTGTTGTGACATT	partial 5′-UTR (-63bp from ATG), exons 1-5	990
#2	F: AGTTTCGTGGACTCGATGTG<br>F: AGTTTCGTGGACTCGATGTG<br>R: ACCTTCCACTGTACTTCATAC	exons 4-11	950
#3	F: CATTTAAAGAACAAATCCATC<br>R: TAAGGAGGATATTTCTAGCAG	exons 9-17	870
#4	F: CTCATACATAGAAAGGGATGT<br>R: TCTGAACTCTTACAACAGGAT	exons 16-21, partial 3′-UTR (201bp from the stop codon)	898
